# Supplementary material for: A Novel Hypoxia-Immune Signature for Gastric Cancer Prognosis and Immunotherapy: Insights from Bulk and Single-Cell RNA-Seq
Source: Curr Issues Mol Biol. 2025 Jul 16;47(7):552. doi: 10.3390/cimb47070552 (PMC12293835; doi:10.3390/cimb47070552)
Supplement: Supplementary file 1 [file cimb-47-00552-s001.zip › cimb-3744695-supplementary.pdf]

Figure S1. a Forest plot presenting the multivariable Cox model results of five genes within the signature [Figures created by R, version 4.1.1.]

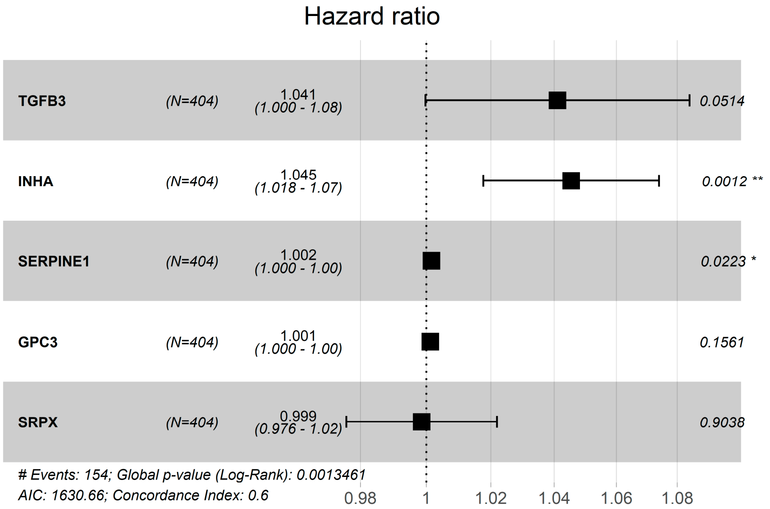

Figure S2. GO annotation and KEGG pathway of 44 hypoxia-immune-related genes. **a** PPI network of the 44-hypoxia-immnue-related genes. **b** KEGG pathway of 44 genes. **c** GO enrichment analysis of 44 hypoxia-immune-related genes

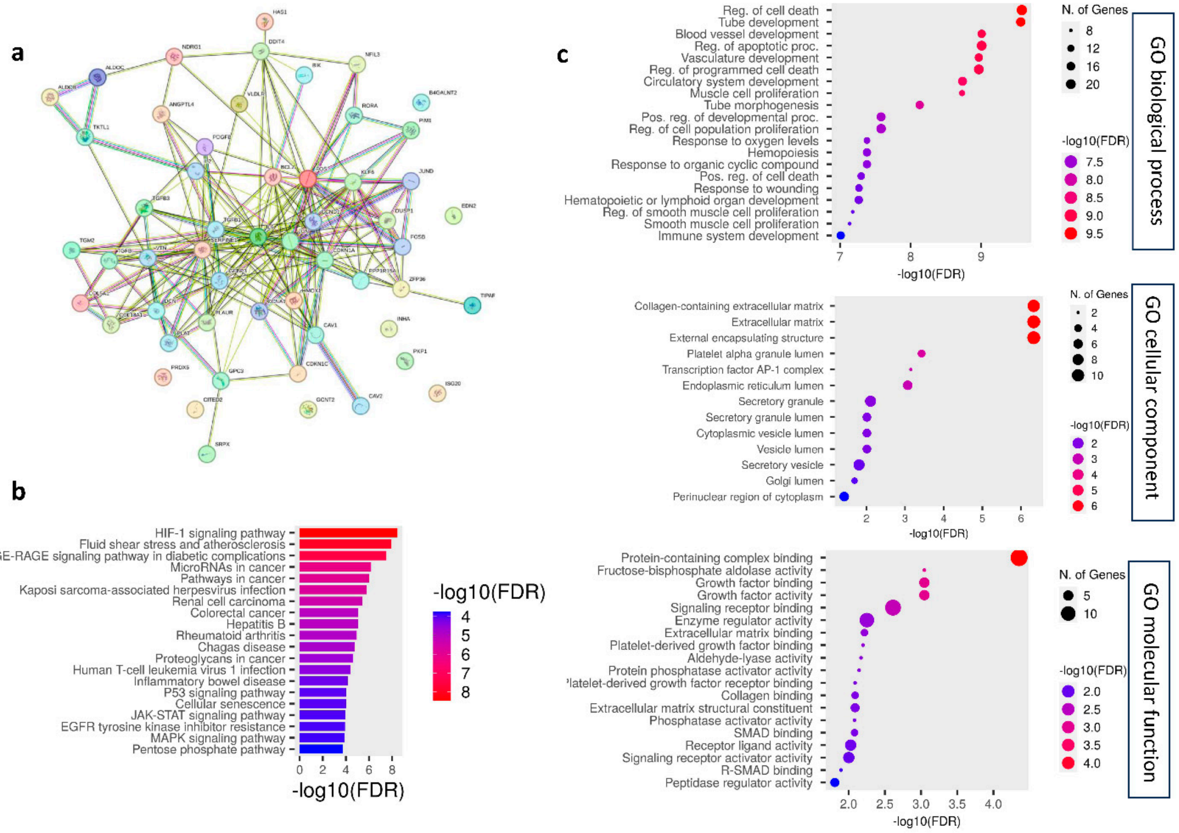

Figure S3. HIF-1 signaling pathway diagram.

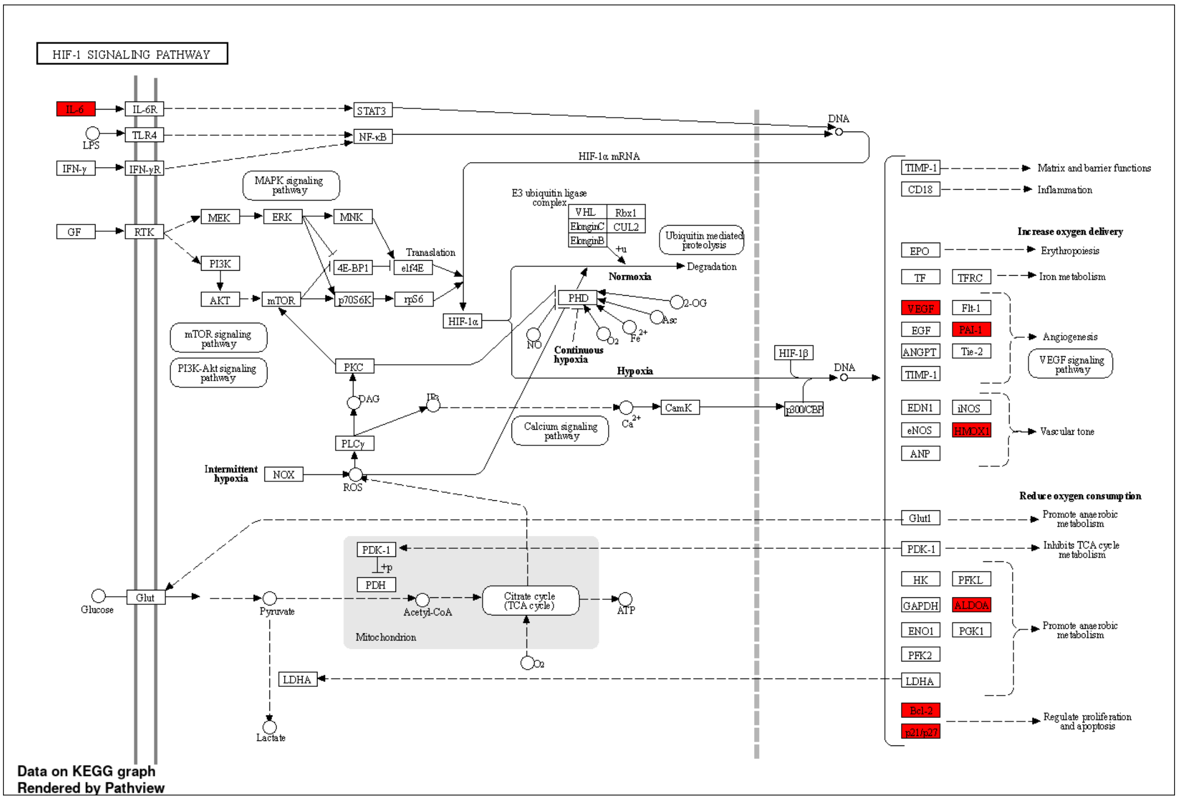

Figure S4. ROC curves of 1-year and 3-year survival prediction of risk score and clinicopathological factors. [Figures created by R, version 4.1.1.]

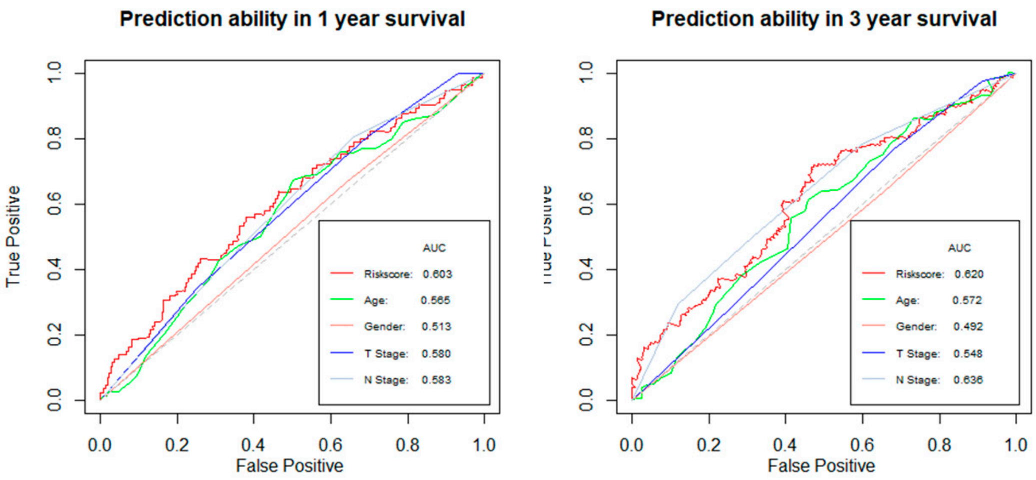

Figure S5. Figures created by R, version 4.1.1.]

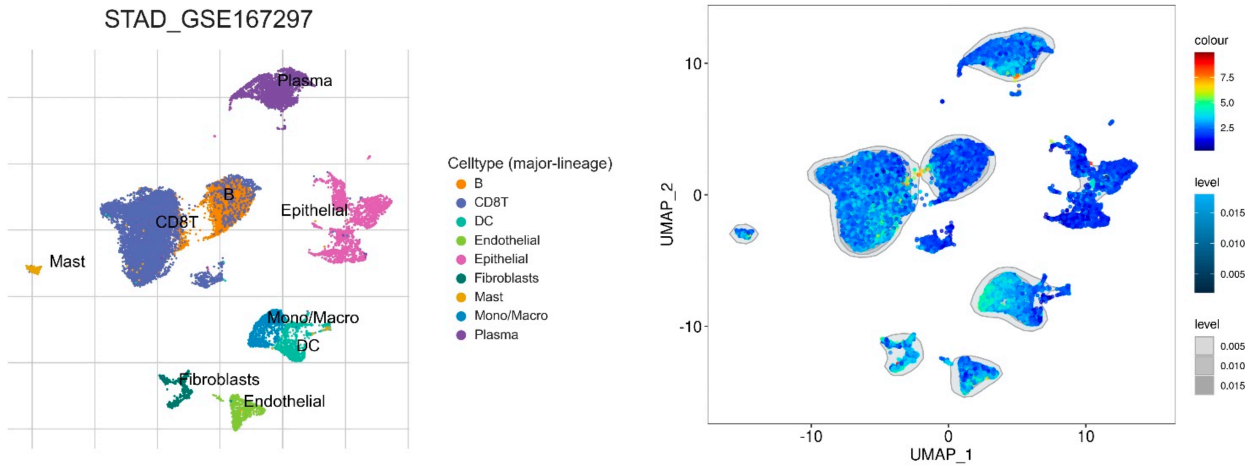

Table S1. Cox regression analysis of 44 hypoxia-immune-related genes

Gene name	HR	HR.95L	HR.95U	*p*-value
INHA	1.048	1.022	1.076	0.000305
TGFB3	1.044	1.019	1.068	0.000357
SERPINE1	1.002	1.001	1.003	0.0048
SRPX	1.013	0.9978	1.029	0.019
GPC3	1.002	0.9999	1.003	0.0422
KLF6	1.007	0.9999	1.015	0.0543
ISG20	0.9628	0.9256	1.001	0.0585
PIM1	1.01	1.001	1.018	0.0709
COL5A1	1.003	0.9997	1.007	0.0741
DUSP1	1.001	1	1.002	0.0846
PRDX5	0.9977	0.995	1	0.103
CAV1	1.002	0.9994	1.004	0.145
PDGFB	1.022	0.991	1.054	0.166
HAS1	1.037	0.979	1.099	0.216
ZFP36	1	0.9998	1.001	0.249
NDRG1	0.9954	0.9872	1.004	0.281
DCN	1.002	0.998	1.006	0.304
CDKN1C	1.004	0.9961	1.011	0.344
IGFBP3	1.001	0.9985	1.004	0.347
TGFBI	1.002	0.9979	1.006	0.374

CDKN1A	1.002	0.9972	1.007	0.401
RORA	1.068	0.902	1.265	0.444
PPP1R15A	1.003	0.995	1.011	0.479
PLAUR	1.004	0.9928	1.015	0.498
FOS	1	0.9993	1.001	0.521
VEGFA	1.004	0.9917	1.016	0.552
ALDOB	1	0.9993	1.001	0.569
IL6	1.004	0.9891	1.02	0.584
PKP1	0.9987	0.994	1.004	0.604
ALDOC	1.009	0.9915	0.9757	0.613
VLDLR	1.042	0.8636	1.257	0.668
BCL2	1.032	0.8908	1.196	0.674
ANGPTL4	1.003	0.9865	1.021	0.693
TIPARP	0.9941	0.9649	1.024	0.697
TKTL1	1.003	0.9845	1.021	0.773
HMOX1	0.9981	0.9851	1.011	0.775
DDIT4	0.9988	0.9903	1.008	0.794
JUN	1	0.9984	1.002	0.81
GCNT2	0.9858	0.8547	1.137	0.844
TGM2	1	0.9976	1.003	0.866
B4GALNT2	1.009	0.8798	1.158	0.895
EDN2	1.004	0.9428	1.069	0.899
NFIL3	0.9992	0.9826	1.016	0.929
CITED2	1	0.9919	1.009	0.933

Table S2. Characteristics of gene signature

Gene ID	Ensemble Gene ID	Entrez	Type	Chromosome	Position	Description	Function
SRPX	ENSG00000101955	8406	protein_coding	X	38.149336	sushi repeat containing protein X-linked [Source:HGNC Symbol;Acc:HGNC:11309]	May be involved in phagocytosis during disk shedding, cell adhesion to cells other than the pigment epithelium or signal transduction.

GPC3	ENSG00000147257	2719	protein_coding	X	133.535745	Glypican-3 alpha subunit	Cell surface proteoglycan that bears heparan sulfate. Negatively regulates the hedgehog signaling pathway when attached via the GPI-anchor to the cell surface by competing with the hedgehog receptor PTC1 for binding to hedgehog proteins (By similarity). Binding to the hedgehog protein SHH triggers internalization of the complex by endocytosis and its subsequent lysosomal degradation (By similarity). Positively regulates the canonical Wnt signaling pathway by binding to the Wnt receptor Frizzled and stimulating the binding of the Frizzled receptor to Wnt ligand [...]
TGFB3	ENSG00000119699	7043	protein_coding	14	75.958097	transforming growth factor beta 3 [Source:HGNC Symbol;Acc:HGNC:11769]	Transforming growth factor beta-3 proprotein: Precursor of the Latency-associated peptide (LAP) and Transforming growth factor beta-3 (TGF-beta-3) chains, which constitute the regulatory and active subunit of TGF-beta-3, respectively. Transforming growth factor beta-3: Multifunctional protein that regulates embryogenesis and cell differentiation and is required in various processes such as secondary palate development (By similarity). Activation into mature form follows different steps: following cleavage of the proprotein in the Golgi appa
INHA	ENSG00000123999	3623	protein_coding	2	219.569162	inhibin subunit alpha [Source:HGNC Symbol;Acc:HGNC:6065]	Inhibins and activins inhibit and activate, respectively, the secretion of follitropin by the pituitary gland. Inhibins/activins are involved in regulating a number of diverse functions such as hypothalamic and pituitary hormone secretion, gonadal hormone secretion, germ cell development and maturation, erythroid differentiation, insulin secretion, nerve cell survival, embryonic axial development or bone growth, depending on their subunit composition. Inhibins appear to oppose the functions of activins; Belongs to the TGF-beta family.

SERPINE1	ENSG00000106366	5054	protein_coding	7	101.127104	serpin family E member 1 [Source:HGNC Symbol;Acc:HGNC:8583],Plasminogen activator inhibitor 1; Serine protease inhibitor. Inhibits TMPRSS7.	Is a primary inhibitor of tissue-type plasminogen activator (PLAT) and urokinase-type plasminogen activator (PLAU). As PLAT inhibitor, it is required for fibrinolysis down-regulation and is responsible for the controlled degradation of blood clots. As PLAU inhibitor, it is involved in the regulation of cell adhesion and spreading. Acts as a regulator of cell migration, independently of its role as protease inhibitor. It is required for stimulation of keratinocyte migration during cutaneous injury repair.
